# Supplementary material for: Pyruvate Dehydrogenase Kinase 1 inhibition mediated oxidative phosphorylation enhancement in cartilage promotes osteoarthritis progression
Source: BMC Musculoskelet Disord. 2023 Jul 20;24:597. doi: 10.1186/s12891-023-06585-6 (PMC10357736; doi:10.1186/s12891-023-06585-6)
Supplement: Supplementary file 1 — Additional file 1. [file 12891_2023_6585_MOESM1_ESM.zip › MF.Gsea.1653627316242/GOMF_ANTIGEN_BINDING.html]

Details for gene set GOMF\_ANTIGEN\_BINDING[GSEA]

|  || Dataset | 55457\_collapsed\_to\_symbols.cls.cls #OA\_versus\_normal.cls.cls #OA\_versus\_normal\_repos |
| Phenotype | cls.cls#OA\_versus\_normal\_repos |
| Upregulated in class | OA |
| GeneSet | GOMF\_ANTIGEN\_BINDING |
| Enrichment Score (ES) | 0.37858656 |
| Normalized Enrichment Score (NES) | 1.7296879 |
| Nominal p-value | 0.0 |
| FDR q-value | 0.07029319 |
| FWER p-Value | 0.793 |
Table: GSEA Results Summary

  

Fig 1: Enrichment plot: GOMF\_ANTIGEN\_BINDING      
 Profile of the Running ES Score & Positions of GeneSet Members on the Rank Ordered List

  

| SYMBOL | TITLE | RANK IN GENE LIST | RANK METRIC SCORE | RUNNING ES | CORE ENRICHMENT || 1 | HFE | homeostatic iron regulator [Source:HGNC Symbol;Acc:HGNC:4886] | 16 | 1.130 | 0.0404 | Yes |
| 2 | MFAP4 | microfibril associated protein 4 [Source:HGNC Symbol;Acc:HGNC:7035] | 365 | 0.719 | 0.0397 | Yes |
| 3 | TSPAN4 | tetraspanin 4 [Source:HGNC Symbol;Acc:HGNC:11859] | 414 | 0.700 | 0.0618 | Yes |
| 4 | IGHG1 | immunoglobulin heavy constant gamma 1 (G1m marker) [Source:HGNC Symbol;Acc:HGNC:5525] | 607 | 0.621 | 0.0696 | Yes |
| 5 | TAPBPL | TAP binding protein like [Source:HGNC Symbol;Acc:HGNC:30683] | 789 | 0.572 | 0.0766 | Yes |
| 6 | FCGRT | Fc fragment of IgG receptor and transporter [Source:HGNC Symbol;Acc:HGNC:3621] | 800 | 0.570 | 0.0968 | Yes |
| 7 | IGKV1D-39 | immunoglobulin kappa variable 1D-39 [Source:HGNC Symbol;Acc:HGNC:5756] | 988 | 0.520 | 0.1014 | Yes |
| 8 | IGLV2-14 | immunoglobulin lambda variable 2-14 [Source:HGNC Symbol;Acc:HGNC:5888] | 1053 | 0.506 | 0.1150 | Yes |
| 9 | IGHV3-72 | immunoglobulin heavy variable 3-72 [Source:HGNC Symbol;Acc:HGNC:5622] | 1062 | 0.504 | 0.1330 | Yes |
| 10 | TRDC | T cell receptor delta constant [Source:HGNC Symbol;Acc:HGNC:12253] | 1100 | 0.496 | 0.1483 | Yes |
| 11 | IGKV2D-28 | immunoglobulin kappa variable 2D-28 [Source:HGNC Symbol;Acc:HGNC:5799] | 1126 | 0.491 | 0.1645 | Yes |
| 12 | IGKV3-20 | immunoglobulin kappa variable 3-20 [Source:HGNC Symbol;Acc:HGNC:5817] | 1154 | 0.486 | 0.1803 | Yes |
| 13 | IGLC2 | immunoglobulin lambda constant 2 [Source:HGNC Symbol;Acc:HGNC:5856] | 1210 | 0.474 | 0.1934 | Yes |
| 14 | HLA-DQA1 | "major histocompatibility complex, class II, DQ alpha 1 [Source:HGNC Symbol;Acc:HGNC:4942]" | 1334 | 0.452 | 0.2005 | Yes |
| 15 | IGHA1 | immunoglobulin heavy constant alpha 1 [Source:HGNC Symbol;Acc:HGNC:5478] | 1426 | 0.439 | 0.2096 | Yes |
| 16 | TRBC1 | T cell receptor beta constant 1 [Source:HGNC Symbol;Acc:HGNC:12156] | 1458 | 0.435 | 0.2232 | Yes |
| 17 | IGKC | immunoglobulin kappa constant [Source:HGNC Symbol;Acc:HGNC:5716] | 1477 | 0.432 | 0.2377 | Yes |
| 18 | ABO | "ABO, alpha 1-3-N-acetylgalactosaminyltransferase and alpha 1-3-galactosyltransferase [Source:HGNC Symbol;Acc:HGNC:79]" | 1495 | 0.429 | 0.2522 | Yes |
| 19 | IGHV3-33 | immunoglobulin heavy variable 3-33 [Source:HGNC Symbol;Acc:HGNC:5596] | 1511 | 0.427 | 0.2667 | Yes |
| 20 | JCHAIN | joining chain of multimeric IgA and IgM [Source:HGNC Symbol;Acc:HGNC:5713] | 1805 | 0.384 | 0.2580 | Yes |
| 21 | ITGA4 | integrin subunit alpha 4 [Source:HGNC Symbol;Acc:HGNC:6140] | 1854 | 0.379 | 0.2682 | Yes |
| 22 | IGLV3-25 | immunoglobulin lambda variable 3-25 [Source:HGNC Symbol;Acc:HGNC:5908] | 1977 | 0.363 | 0.2721 | Yes |
| 23 | CD1E | CD1e molecule [Source:HGNC Symbol;Acc:HGNC:1638] | 2038 | 0.357 | 0.2806 | Yes |
| 24 | CD48 | CD48 molecule [Source:HGNC Symbol;Acc:HGNC:1683] | 2082 | 0.352 | 0.2902 | Yes |
| 25 | IGKV4-1 | immunoglobulin kappa variable 4-1 [Source:HGNC Symbol;Acc:HGNC:5834] | 2132 | 0.346 | 0.2991 | Yes |
| 26 | IGHM | immunoglobulin heavy constant mu [Source:HGNC Symbol;Acc:HGNC:5541] | 2241 | 0.335 | 0.3030 | Yes |
| 27 | IGHV3-21 | immunoglobulin heavy variable 3-21 [Source:HGNC Symbol;Acc:HGNC:5586] | 2404 | 0.314 | 0.3019 | Yes |
| 28 | IGLV3-19 | immunoglobulin lambda variable 3-19 [Source:HGNC Symbol;Acc:HGNC:5903] | 2440 | 0.309 | 0.3106 | Yes |
| 29 | LILRA2 | leukocyte immunoglobulin like receptor A2 [Source:HGNC Symbol;Acc:HGNC:6603] | 2444 | 0.309 | 0.3217 | Yes |
| 30 | IGHV1-69 | immunoglobulin heavy variable 1-69 [Source:HGNC Symbol;Acc:HGNC:5558] | 2461 | 0.307 | 0.3318 | Yes |
| 31 | TGFB1 | transforming growth factor beta 1 [Source:HGNC Symbol;Acc:HGNC:11766] | 2490 | 0.303 | 0.3408 | Yes |
| 32 | LAG3 | lymphocyte activating 3 [Source:HGNC Symbol;Acc:HGNC:6476] | 2682 | 0.282 | 0.3363 | Yes |
| 33 | CD1B | CD1b molecule [Source:HGNC Symbol;Acc:HGNC:1635] | 2697 | 0.279 | 0.3455 | Yes |
| 34 | IGHV4-61 | immunoglobulin heavy variable 4-61 [Source:HGNC Symbol;Acc:HGNC:5655] | 2722 | 0.278 | 0.3538 | Yes |
| 35 | IGHV3-7 | immunoglobulin heavy variable 3-7 [Source:HGNC Symbol;Acc:HGNC:5620] | 2798 | 0.270 | 0.3579 | Yes |
| 36 | HLA-DQB1 | "major histocompatibility complex, class II, DQ beta 1 [Source:HGNC Symbol;Acc:HGNC:4944]" | 2840 | 0.266 | 0.3645 | Yes |
| 37 | IGHV3-73 | immunoglobulin heavy variable 3-73 [Source:HGNC Symbol;Acc:HGNC:5623] | 3082 | 0.241 | 0.3546 | Yes |
| 38 | DHCR24 | 24-dehydrocholesterol reductase [Source:HGNC Symbol;Acc:HGNC:2859] | 3126 | 0.237 | 0.3600 | Yes |
| 39 | TRGV3 | T cell receptor gamma variable 3 [Source:HGNC Symbol;Acc:HGNC:12288] | 3137 | 0.236 | 0.3679 | Yes |
| 40 | TOPORS | "TOP1 binding arginine/serine rich protein, E3 ubiquitin ligase [Source:HGNC Symbol;Acc:HGNC:21653]" | 3244 | 0.227 | 0.3680 | Yes |
| 41 | CD1C | CD1c molecule [Source:HGNC Symbol;Acc:HGNC:1636] | 3354 | 0.216 | 0.3675 | Yes |
| 42 | TRBC2 | T cell receptor beta constant 2 [Source:HGNC Symbol;Acc:HGNC:12157] | 3401 | 0.211 | 0.3717 | Yes |
| 43 | IGHV3-23 | immunoglobulin heavy variable 3-23 [Source:HGNC Symbol;Acc:HGNC:5588] | 3449 | 0.207 | 0.3756 | Yes |
| 44 | CD1D | CD1d molecule [Source:HGNC Symbol;Acc:HGNC:1637] | 3516 | 0.201 | 0.3779 | Yes |
| 45 | SIRPA | signal regulatory protein alpha [Source:HGNC Symbol;Acc:HGNC:9662] | 3599 | 0.193 | 0.3786 | Yes |
| 46 | TAPBP | TAP binding protein [Source:HGNC Symbol;Acc:HGNC:11566] | 4527 | 0.111 | 0.3104 | No |
| 47 | IGHV4-34 | immunoglobulin heavy variable 4-34 [Source:HGNC Symbol;Acc:HGNC:5650] | 4746 | 0.091 | 0.2967 | No |
| 48 | KLRD1 | killer cell lectin like receptor D1 [Source:HGNC Symbol;Acc:HGNC:6378] | 4778 | 0.089 | 0.2976 | No |
| 49 | CD1A | CD1a molecule [Source:HGNC Symbol;Acc:HGNC:1634] | 5142 | 0.060 | 0.2715 | No |
| 50 | LILRA1 | leukocyte immunoglobulin like receptor A1 [Source:HGNC Symbol;Acc:HGNC:6602] | 5328 | 0.046 | 0.2587 | No |
| 51 | HLA-DPB1 | "major histocompatibility complex, class II, DP beta 1 [Source:HGNC Symbol;Acc:HGNC:4940]" | 5486 | 0.033 | 0.2477 | No |
| 52 | SLAMF1 | signaling lymphocytic activation molecule family member 1 [Source:HGNC Symbol;Acc:HGNC:10903] | 5823 | 0.007 | 0.2217 | No |
| 53 | IGHV5-51 | immunoglobulin heavy variable 5-51 [Source:HGNC Symbol;Acc:HGNC:5659] | 6399 | -0.035 | 0.1782 | No |
| 54 | HLA-DRA | "major histocompatibility complex, class II, DR alpha [Source:HGNC Symbol;Acc:HGNC:4947]" | 6550 | -0.046 | 0.1681 | No |
| 55 | CD209 | CD209 molecule [Source:HGNC Symbol;Acc:HGNC:1641] | 6600 | -0.050 | 0.1661 | No |
| 56 | TAP1 | "transporter 1, ATP binding cassette subfamily B member [Source:HGNC Symbol;Acc:HGNC:43]" | 6633 | -0.052 | 0.1656 | No |
| 57 | CD40 | CD40 molecule [Source:HGNC Symbol;Acc:HGNC:11919] | 6825 | -0.065 | 0.1530 | No |
| 58 | KLRC1 | killer cell lectin like receptor C1 [Source:HGNC Symbol;Acc:HGNC:6374] | 6835 | -0.066 | 0.1548 | No |
| 59 | IGHV3-20 | immunoglobulin heavy variable 3-20 [Source:HGNC Symbol;Acc:HGNC:5585] | 6859 | -0.068 | 0.1555 | No |
| 60 | SPON2 | spondin 2 [Source:HGNC Symbol;Acc:HGNC:11253] | 7001 | -0.080 | 0.1474 | No |
| 61 | HLA-DRB1 | "major histocompatibility complex, class II, DR beta 1 [Source:HGNC Symbol;Acc:HGNC:4948]" | 7087 | -0.086 | 0.1440 | No |
| 62 | HLA-F | "major histocompatibility complex, class I, F [Source:HGNC Symbol;Acc:HGNC:4963]" | 7196 | -0.096 | 0.1391 | No |
| 63 | MAML1 | mastermind like transcriptional coactivator 1 [Source:HGNC Symbol;Acc:HGNC:13632] | 7275 | -0.101 | 0.1367 | No |
| 64 | CLEC4M | C-type lectin domain family 4 member M [Source:HGNC Symbol;Acc:HGNC:13523] | 7455 | -0.115 | 0.1270 | No |
| 65 | IGLL1 | immunoglobulin lambda like polypeptide 1 [Source:HGNC Symbol;Acc:HGNC:5870] | 7544 | -0.122 | 0.1246 | No |
| 66 | FCN3 | ficolin 3 [Source:HGNC Symbol;Acc:HGNC:3625] | 9175 | -0.246 | 0.0065 | No |
| 67 | IL7R | interleukin 7 receptor [Source:HGNC Symbol;Acc:HGNC:6024] | 9233 | -0.251 | 0.0113 | No |
| 68 | EP400 | E1A binding protein p400 [Source:HGNC Symbol;Acc:HGNC:11958] | 9304 | -0.256 | 0.0152 | No |
| 69 | IGHD | immunoglobulin heavy constant delta [Source:HGNC Symbol;Acc:HGNC:5480] | 9396 | -0.263 | 0.0178 | No |
| 70 | HLA-DPA1 | "major histocompatibility complex, class II, DP alpha 1 [Source:HGNC Symbol;Acc:HGNC:4938]" | 9481 | -0.273 | 0.0213 | No |
| 71 | SLC7A9 | solute carrier family 7 member 9 [Source:HGNC Symbol;Acc:HGNC:11067] | 9539 | -0.277 | 0.0271 | No |
| 72 | FCN1 | ficolin 1 [Source:HGNC Symbol;Acc:HGNC:3623] | 10180 | -0.339 | -0.0104 | No |
| 73 | SLC7A8 | solute carrier family 7 member 8 [Source:HGNC Symbol;Acc:HGNC:11066] | 10228 | -0.344 | -0.0014 | No |
| 74 | PPP2R1A | protein phosphatase 2 scaffold subunit Aalpha [Source:HGNC Symbol;Acc:HGNC:9302] | 10395 | -0.360 | -0.0011 | No |
| 75 | IGHE | immunoglobulin heavy constant epsilon [Source:HGNC Symbol;Acc:HGNC:5522] | 10676 | -0.390 | -0.0085 | No |
| 76 | HLA-G | "major histocompatibility complex, class I, G [Source:HGNC Symbol;Acc:HGNC:4964]" | 10869 | -0.413 | -0.0083 | No |
| 77 | FCN2 | ficolin 2 [Source:HGNC Symbol;Acc:HGNC:3624] | 10937 | -0.423 | 0.0021 | No |
| 78 | PLG | plasminogen [Source:HGNC Symbol;Acc:HGNC:9071] | 10939 | -0.423 | 0.0176 | No |
| 79 | GP2 | glycoprotein 2 [Source:HGNC Symbol;Acc:HGNC:4441] | 11217 | -0.459 | 0.0129 | No |
| 80 | TAP2 | "transporter 2, ATP binding cassette subfamily B member [Source:HGNC Symbol;Acc:HGNC:44]" | 11616 | -0.519 | 0.0010 | No |
| 81 | HLA-E | "major histocompatibility complex, class I, E [Source:HGNC Symbol;Acc:HGNC:4962]" | 11659 | -0.526 | 0.0171 | No |
| 82 | SLC7A5 | solute carrier family 7 member 5 [Source:HGNC Symbol;Acc:HGNC:11063] | 12203 | -0.646 | -0.0015 | No |
| 83 | HLA-A | "major histocompatibility complex, class I, A [Source:HGNC Symbol;Acc:HGNC:4931]" | 12502 | -0.749 | 0.0028 | No |
| 84 | HLA-B | "major histocompatibility complex, class I, B [Source:HGNC Symbol;Acc:HGNC:4932]" | 12534 | -0.764 | 0.0286 | No |
Table: GSEA details [plain text format]

  

Fig 2: GOMF\_ANTIGEN\_BINDING      
 Blue-Pink O' Gram in the Space of the Analyzed GeneSet

  

Fig 3: GOMF\_ANTIGEN\_BINDING: Random ES distribution      
 Gene set null distribution of ES for **GOMF\_ANTIGEN\_BINDING**
